# Venous air embolism induced by burr hole drilling before dural incision in craniotomy: two case reports

**DOI:** 10.1186/s40981-025-00823-7

**Published:** 2025-10-22

**Authors:** Yohei Motoi, Shuji Okahara, Makiko Tani, Nobushige Tsuboi, Hiroshi Morimatsu

**Affiliations:** 1 Department of Anesthesiology and Resuscitology, Okayama University Hospital, 2-5-1 Shikatacho, Kitaku, Okayama City, Okayama Japan; 2 Department of Neurosurgery, Okayama Red Cross Hospital, 2-1-1 Aoe, Kita-ku, Okayama, 700-8607 Japan

**Keywords:** Venous air embolism, Transesophageal echocardiography, Computed tomography, Diploic veins, Emissary veins, Burr hole drilling, Case report

## Abstract

**Background:**

Venous air embolism (VAE) is a rare but potentially fatal complication in neurosurgery typically caused by injury to dura mater, especially venous sinuses, during craniotomy. We report two cases of VAE that occurred before dural incision.

**Case presentation:**

Both patients underwent craniotomy under general anesthesia in a head-up position. Hemodynamic and respiratory deterioration occurred during or immediately after burr hole drilling with abnormal vital signs and transesophageal echocardiography findings, raising suspicion for VAE. Immediate management, including surgical field protection and cardiopulmonary support, stabilized the patients’ conditions. The procedure was subsequently discontinued in case 1 and modified to limited resection in case 2. Postoperative computed tomography revealed intracranial venous air within the internal jugular vein, cavernous sinus, and diploic veins.

**Conclusion:**

These cases highlight that VAE can occur even before dural incision. Vigilant intraoperative monitoring and prompt intervention are essential for preventing potentially fatal outcomes.

## Background

Venous air embolism (VAE) can occur during surgery or interventional procedures or following thoracic trauma [1]. Neurosurgical operations carry a high risk of VAE, especially when performed with patients in a head-elevated position [2]. VAE during craniotomy is typically caused by air entering major cerebral venous sinuses in a head-elevated position, most often after dural incision [3]. VAE severity is determined on the basis of the rate and volume of air entering the venous system; therefore, prompt diagnosis and treatment are essential [4].

We report two cases of VAE diagnosed intraoperatively through the vigilant monitoring of vital signs and transesophageal echocardiography (TEE), with postoperative confirmation using computed tomography (CT) scans. Although cerebral venous sinus injury is a well-known trigger for VAE during craniotomy, typically following dural incision, both patients demonstrated VAE at an atypical stage, during or immediately after burr hole drilling, prior to dural incision. These findings highlight the importance of vigilance during early stages of neurosurgical procedures.

## Case presentation

### Case 1

**Fig. 1:**
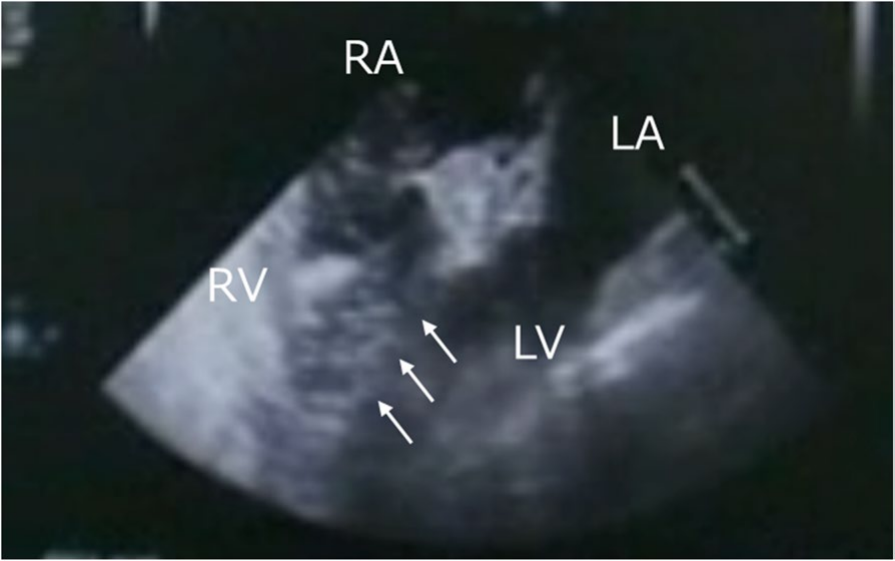
Intraoperative transesophageal echocardiography findings in case 1. Four-chamber mid-esophageal echocardiographic image showing air bubble shadows in the right ventricle (arrows). LA: left atrium; LV: left ventricle; RA: right atrium; RV: right ventricle

**Fig. 2:**
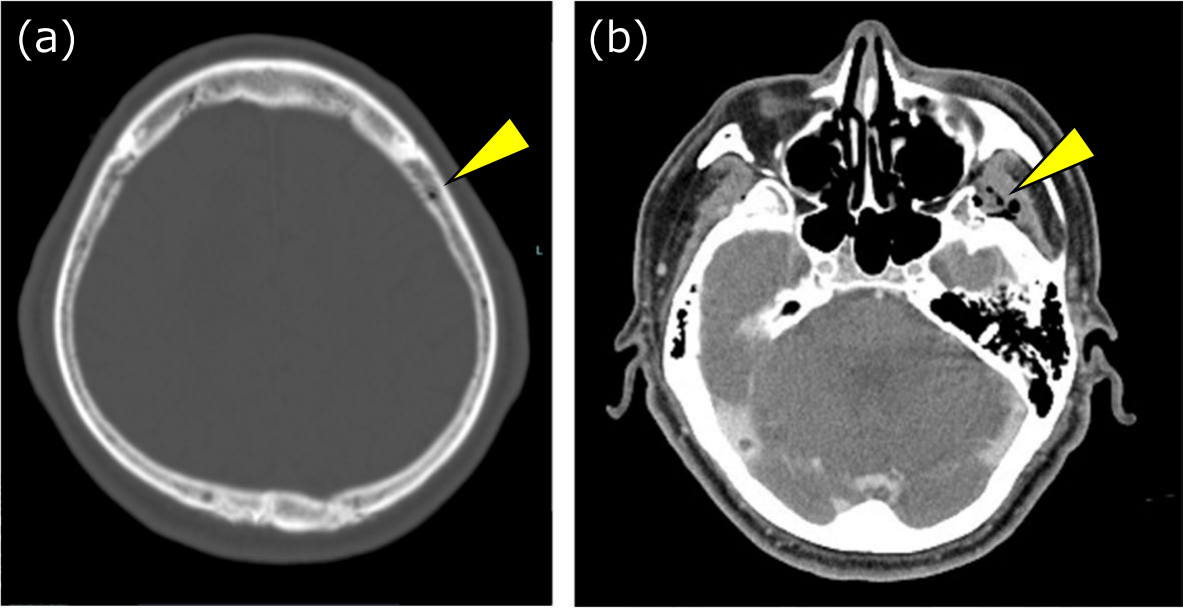
Postoperative CT findings in case 1. **a** CT brain image showing air densities in the diploic layer of the skull. **b** CT brain image showing air densities in the bilateral cervical regions

A 63-year-old man underwent craniotomy for biopsy of a lesion in the right frontal lobe. He had no specific medical history or abnormal laboratory findings except for the prophylactic use of levetiracetam. General anesthesia was induced with propofol, rocuronium, and remifentanil, and the patient was hemodynamically stable. Surgery was performed with the patient positioned in an approximately 20° head-up tilt. Shortly after burr hole drilling with an electric cranial drill, there was a sudden decrease in the end-tidal partial pressure of carbon dioxide (P_ET_CO_2_) from 36 to 24 mmHg. This was followed by a drop in the saturation of percutaneous oxygen (SpO_2_) from 98 to 94% and blood pressure from 106/69 to 80/52 mmHg. Arterial blood gas analysis revealed a decreased partial pressure of arterial oxygen (PaO_2_) of 113 mmHg with a 100% fraction of inspired oxygen (FiO_2_) and a partial pressure of carbon dioxide (PaCO_2_) of 53 mmHg, indicating a significant discrepancy between P_ET_CO_2_ and PaCO_2_. Suspecting VAE, we repositioned the patient in a horizontal position, ventilated him with 100% oxygen, covered the surgical field with saline-soaked gauze, and initiated fluid resuscitation and vasopressor therapy. Transesophageal echocardiography (TEE) was immediately performed, revealing air bubbles in the right ventricle (Fig. 1). Although oxygenation improved (PaO_2_/FiO_2_ = 311), the discrepancy between P_ET_CO_2_ and PaCO_2_ persisted. After discussion with the surgical team, the incision was promptly closed and the patient was transported for a postoperative CT scan. Contrast-enhanced chest CT revealed no air or thrombi within the pulmonary arteries. Instead, evidence of air density was observed within the soft tissue of the bilateral cervical regions and diploic layer of the skull (Fig. 2).

### Case 2

**Fig. 3:**
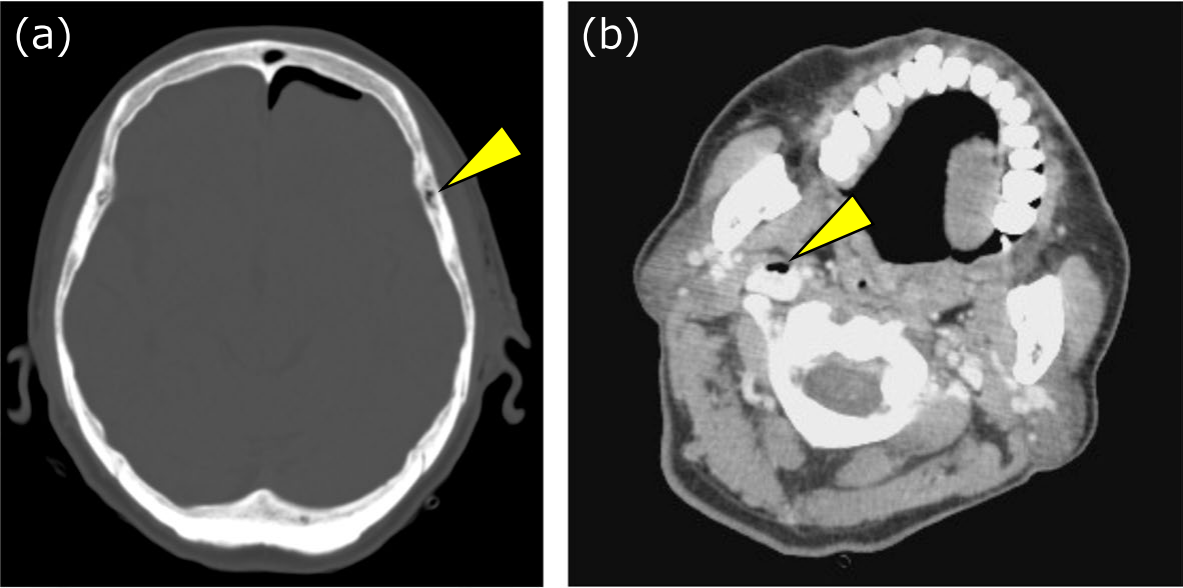
Postoperative CT findings in case 2. **a** CT brain image showing air densities in the diploic layer of the skull. **b** CT brain image showing air densities in the right internal jugular vein

A 70-year-old woman underwent craniotomy for brain tumor resection. She had a history of hypertension and type 2 diabetes mellitus and was taking linagliptin, nifedipine, enalapril maleate, and levetiracetam. The preoperative tests revealed no significant abnormalities. General anesthesia was induced using propofol, rocuronium, and remifentanil. Surgery was started with approximately a 30° head-up tilt. During burr hole drilling, a sudden decrease in P_ET_CO_2_ from 32 to 25 mmHg was observed, along with a drop in SpO_2_ from 100 to 87% and in blood pressure from 128/60 to 80/45 mmHg. Arterial blood gas analysis revealed PaCO_2_ of 47 mmHg, indicating a significant PaCO_2_–P_ET_CO_2_ discrepancy. Immediate interventions for preventing further air entry by repositioning the patient, covering the surgical field, and administering 100% oxygen along with fluid resuscitation and vasopressors led to the stabilization of vital signs and improvement in the PaCO_2_–P_ET_CO_2_ discrepancy. Although TEE was initially considered, the patient’s head and neck positioning (in a fixed, flexed posture), with the surgical field located in close proximity to the oral cavity, rendered TEE insertion technically difficult and potentially hazardous. Considering the risk of oropharyngeal or esophageal injury associated with TEE under these conditions, we decided not to proceed. Instead, transthoracic echocardiography (TTE) was performed, showing no evidence of intracardiac air or right heart strain. Given the patient’s stabilized circulatory and respiratory status after treatment, the surgical plan was revised to partial resection, and the procedure was continued. Postoperative contrast-enhanced chest CT revealed no evidence of air or thrombi in the pulmonary arteries. However, head and neck CT showed the presence of air within the diploic veins of the skull, as well as in the right internal jugular vein and cavernous sinus, which had not been observed preoperatively (Fig. 3).

Both patients were transferred to the intensive care unit (ICU) under sedation and intubation, with no worsening of hypoxemia observed thereafter. The patients were extubated on the day following surgery, had an uneventful recovery without complications such as paralysis, and were subsequently discharged from the ICU.

## Discussion

In these cases, VAE was suspected on the basis of sudden decreases in P_ET_CO_2_, SpO_2_, and blood pressure during and immediately after burr hole drilling prior to dural incision. It was later confirmed through postoperative CT scans, which revealed residual air in the diploic and internal jugular veins that had not been observed preoperatively. These findings suggest that even in the absence of dural or venous sinus injury, the risk of VAE remains significant during the early stages of neurosurgical procedures.

**Fig. 4:**
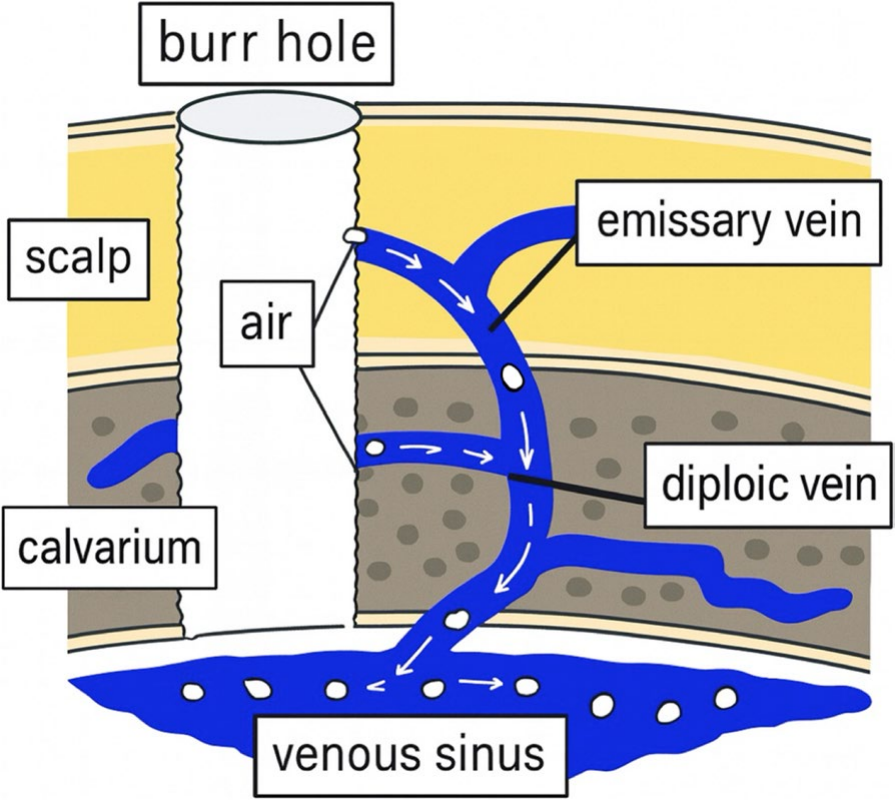
Schematic illustration of the air entry pathway from the burr hole to the intracranial venous system. The emissary vein extends from the scalp to the skull, while the diploic vein courses through the diploic (interplanar) layer and drains into the dural venous sinus

There are two types of veins that drain from the scalp into the skull: emissary veins, which connect the extracranial and intracranial venous systems; and diploic veins, which traverse the cancellous bone layer between the outer and inner plates of the skull and drain into the venous sinus [5] (Fig. 4). The outer walls of the veins are anchored to the surrounding bone tissue, preventing their lumens from collapsing upon injury and facilitating air entry into the venous system, particularly in the head-up position [6]. Neither of these cases had any specific anatomical that would predispose them to VAE, suggesting that such events may occur in any patient under similar conditions. In both cases, electric drills were used instead of air drills. Most cranial electric drills are designed with pressure-release mechanisms and debris evacuation channels that direct bone dust outward during drilling. Therefore, it is unlikely that the pressure generated during burr hole creation with a standard electric drill directly contributed to the development of VAE. Some cases of VAE attributed to air inflow from these veins have been reported following the removal of the Mayfield head fixation device [7, 8] and the placement of Raney clips after scalp incisions [9]. In the present patients, it is likely that the minor tracts created during burr hole drilling allowed air entry. Postoperative head CT revealed air not only in the internal jugular vein and cavernous sinus but also within the diploic veins of the skull. These findings suggest that air entered the cranial venous system from the surgical site, emphasizing the potential risk of VAE before dural incision.

VAE was suspected in these cases on the basis of the following findings: (1) a decrease in P_ET_CO_2_ with a corresponding increase in PaCO_2_, indicating a significant discrepancy; (2) a decrease in SpO_2_; and (3) hypotension. Early diagnosis of VAE requires monitoring devices with high sensitivity and specificity, rapid response, the ability to quantitatively evaluate air embolism, and recovery assessment [10]. TEE provides high sensitivity and specificity for detecting air, even in small volumes, and is valuable for monitoring recovery progress [11, 12]. However, the insertion of a TEE probe can be challenging in certain patient positions such as the prone position. Moreover, there is a risk of injury to the oral cavity or esophagus, particularly in cases involving neck flexion. In case 1, VAE was confirmed using TEE, which revealed high echoic signals suggestive of air in the right ventricle. In contrast, in case 2, TTE revealed neither intracardiac air nor signs of right-sided heart strain. Given the relatively rapid improvement in the patient’s overall condition following circulatory management, including fluid resuscitation, vasopressor administration, and respiratory support, TEE was not performed because the insertion was technically challenging and posed a risk of complications owing to its invasiveness.

In both cases, immediate intraoperative interventions were critical to stabilize the patients. These included repositioning from a head-up tilt to a horizontal position, administration of 100% oxygen, prompt fluid resuscitation, and administration of vasopressors. Saline-soaked gauze was applied to the surgical field to minimize the risk of further air entrainment. Therefore, the patients’ vital signs—including the PaCO_2_–P_ET_CO_2_ discrepancy—showed improvement. Although P_ET_CO_2_ monitoring is not sufficiently sensitive for the early detection of VAE, particularly in cases involving small volumes of air, it was useful in assessing recovery and estimating VAE severity.

Our cases highlight the possibility of fatal VAE even before dural incision. When hypotension and hypoxemia with a sudden decrease in P_ET_CO_2_ are observed, VAE should be suspected even before dural incision. Intraoperative diagnosis and intervention were primarily guided using TEE, whereas postoperative CT provided critical information to rule out pulmonary thromboembolism and confirmed the presence of air in the head and neck regions.

## Data Availability

All data are included in this article.

## Abbreviations

VAE: Venous air embolism
P_ET_CO_2_: End-tidal partial pressure of carbon dioxide
TEE: Transesophageal echocardiography
CT: Computed tomography
SpO_2_: Saturation of percutaneous oxygen
PaO_2_: Partial pressure of arterial oxygen
FiO_2_: Fraction of inspired oxygen
PaCO_2_: Pressure of carbon dioxide
TTE: Transthoracic echocardiography
ICU: Intensive care unit

## References

[1] Palmon SC, Moore LE, Lundberg J, Toung T. Venous air embolism: a review. J Clin Anesth. 1997;9:251–7. 9172037 10.1016/s0952-8180(97)00024-x

[2] Himes BT, Mallory GW, Abcejo AS, Pasternak J, Atkinson JLD, Meyer FB, et al. Contemporary analysis of the intraoperative and perioperative complications of neurosurgical procedures performed in the sitting position. J Neurosurg. 2017;127:182–8. 27494821 10.3171/2016.5.JNS152328

[3] Lee CC, Wu A, Li M. Venous air embolism during neurosurgery. In: Brambrink A, Kirsch J, editors. Essentials of neurosurgical anesthesia & critical care. Cham: Springer; 2020. p. 287–91.

[4] Mirski Marek A, Lele Abhijit V, Fitzsimmons L, Toung Thomas JK, Warltier DC. Diagnosis and treatment of vascular air embolism. Anesthesiology. 2007;106:164–77. 17197859 10.1097/00000542-200701000-00026

[5] Lachkar S, Dols MM, Ishak B, Iwanaga J, Tubbs RS. The diploic veins: a comprehensive review with clinical applications. Cureus. 2019;11:e4422. 10.7759/cureus.4422. 31245209 10.7759/cureus.4422 PMC6559436

[6] Tufegdzic B, Lamperti M, Siyam A, Roser F. Air-embolism in the semi-sitting position for craniotomy: a narrative review with emphasis on a single centers experience. Clin Neurol Neurosurg. 2021. 10.1016/j.clineuro.2021.106904. 34482115 10.1016/j.clineuro.2021.106904

[7] Prabhakar H, Ali Z, Bhagat H. Venous air embolism arising after removal of Mayfield skull clamp. J Neurosurg Anesthesiol. 2008;20:158–9. 18362787 10.1097/ANA.0b013e31816726c4

[8] Grinberg F, Slaughter TF, McGrath BJ. Probable venous air embolism associated with removal of the Mayfield skull clamp. Anesth Analg. 1995;80:1049–50. 7726406 10.1097/00000539-199505000-00036

[9] Spence NZ, Faloba K, Sonabend AM, Bruce JN, Anastasian ZH. Venous air embolus during scalp incision. J Clin Neurosci. 2016;28:170–211. 26765767 10.1016/j.jocn.2015.11.019

[10] Shaikh N, Ummunisa F. Acute management of vascular air embolism. J Emerg Trauma Shock. 2009;2:180–5. 20009308 10.4103/0974-2700.55330 PMC2776366

[11] Souders JE. Pulmonary air embolism. J Clin Monit Comput. 2000;16:375–83. 12580220 10.1023/a:1011455701892

[12] Furuya H, Suzuki T, Okumura F, Kishi Y, Uefuji T. Detection of air embolism by transesophageal echocardiography. Anesthesiology. 1983;58:124–9. 6401948 10.1097/00000542-198302000-00004

